# Performance and Mechanism of Photoelectrocatalytic Activity of MoS*_x_*/WO_3_ Heterostructures Obtained by Reactive Pulsed Laser Deposition for Water Splitting

**DOI:** 10.3390/nano10050871

**Published:** 2020-04-30

**Authors:** Vyacheslav Fominski, Roman Romanov, Dmitry Fominski, Alexey Soloviev, Oxana Rubinkovskaya, Maxim Demin, Ksenia Maksimova, Pavel Shvets, Aleksandr Goikhman

**Affiliations:** 1 National Research Nuclear University MEPhI (Moscow Engineering Physics Institute), Kashirskoe Sh. 31, 115409 Moscow, Russia; limpo2003@mail.ru (R.R.); dmitryfominski@gmail.com (D.F.); ale7@inbox.lv (A.S.); oxygenofunt@gmail.com (O.R.); 2 REC “Functional Nanomaterials”, Immanuel Kant Baltic Federal University, Aleksandra Nevskogo St. 14, 236041 Kaliningrad, Russia; mdemin@kantiana.ru (M.D.); xmaksimova@gmail.com (K.M.); pshvets@kantiana.ru (P.S.); aygoikhman@gmail.com (A.G.)

**Keywords:** reactive pulsed laser deposition, molybdenum sulfides, tungsten oxide, photocatalysis, heterostructure, water splitting

## Abstract

This work studies the factors that affect the efficiency of the photoelectrochemical hydrogen evolution reaction (HER) using MoS*_x_*/WO_3_ nano-heterostructures obtained by reactive pulsed laser deposition (RPLD) on glass substrates covered with fluorinated tin oxide (FTO). Another focus of the research is the potential of MoS*_x_* nanofilms as a precursor for MoO*_z_*(S) nanofilms, which enhance the efficiency of the photo-activated oxygen evolution reaction (OER) using the MoO*_z_*(S)/WO_3_/FTO heterostructures. The nanocrystalline WO_3_ film was created by laser ablation of a W target in dry air at a substrate temperature of 420 °C. Amorphous MoS*_x_* nanofilms (2 ≤ *x* ≤ 12) were obtained by laser ablation of an Mo target in H_2_S gas of varied pressure at room temperature of the substrate. Studies of the energy band structures showed that for all MoS*_x_*/WO_3_/FTO samples, photo-activated HER in an acid solution proceeded through the Z-scheme. The highest photoelectrochemical HER efficiency (a photocurrent density ~1 mA/cm^2^ at a potential of ~0 V under Xe lamp illumination (~100 mW/cm^2^)) was found for porous MoS_4.5_ films containing the highest concentration of catalytically active sites attributed to S ligands. During the anodic posttreatment of porous MoS*_x_* nanofilms, MoO*_z_*(S) films with a narrow energy band gap were formed. The highest OER efficiency (a photocurrent density ~5.3 mA/cm^2^ at 1.6 V) was detected for MoO*_z_*(S)/WO_3_/FTO photoanodes that were prepared by posttreatment of the MoS*_x_*_~3.2_ precursor. The MoO*_z_*(S) film contributed to the effective photogeneration of electron–hole pairs that was followed by the transport of photoelectrons from MoO*_z_*(S) into the WO_3_ film and the effective participation of holes possessing strong oxidation ability in the OER on the surface of the MoO*_z_*(S) film.

## 1. Introduction

Obtaining and studying new nanomaterials for the formation of hybrid- and hetero-structures that provide effective splitting of water to produce hydrogen and oxygen is a principal task of alternative energy [1,2,3]. When creating photoelectrodes based on advanced semiconductor materials for photoelectrochemical cells, it is necessary to take into account a number of factors that affect the efficiency of water splitting. Among these factors are the optical properties of nanomaterials, their electronic structure, and the catalytic properties for activating the evolution of hydrogen and oxygen in aqueous solutions [4,5,6,7]. In the formation of hybrid/nanocomposite and hetero/multilayer structures, these factors affect the generation and transfer of non-equilibrium charge carriers (photogenerated electrons/holes) in semiconductors, the lifetime of these carriers, and the efficiency of catalytically activated processes of water splitting [8,9,10].

Tungsten oxide (WO_3_) is well known as a semiconducting photoactive material that has proved its effectiveness, first of all, for activating the oxygen evolution reaction (OER) [11,12,13,14]. WO_3_-based photoanodes, as well as photoanodes with other promising metal oxides, can be obtained by pulsed laser deposition (PLD). Variation/optimization of the PLD conditions makes it possible to obtain metal oxide photoanodes for photoelectrochemical water splitting with enhanced functional characteristics [15,16,17]. The use of this material to activate the hydrogen evolution reaction (HER) is constrained by the complexity of the formation of a specific structural and chemical state in WO_3_, which has high catalytic activity [18,19]. For this reason, various hybrid- and hetero-structures based on tungsten oxide are currently being developed [20,21,22,23,24,25]. In these structures, the (photo-) electrocatalytic activity of tungsten oxide is enhanced by the deposition of films (co-catalysts) that have a positive effect both on the efficiency of the electrocatalytic process of hydrogen evolution and on the processes of generation and transport of non-equilibrium carriers in a hybrid- or hetero-structure. Previously, Fominski et al. [20] showed that the efficiency of the photoelectrocatalytic HER process on WO_3−*y*_ porous films can be significantly increased by the deposition of a MoS*_x_*_~3_/np-Mo nanofilm. MoS_3_/np-Mo films were produced by PLD from a MoS_2_ target in a buffer gas. Although the use of gas reduced the growth rate of MoS_3_/np-Mo films significantly, it ensured the formation of the original nanostructure, including the amorphous MoS_3_ phase and Mo nanoparticles. WO_3−*y*_ films were created by the PLD method at room temperature of a substrate covered with fluorinated tin oxide (FTO). Thermal annealing of these films in air at 450 °C caused their crystallization, provided the porous morphology of the films did not change. The effect of annealing on the efficiency of the photoelectrocatalytic HER reaction using the (MoS_3_/np-Mo)/WO_3−*y*_/ FTO structures turned out to be ambiguous.

The use of nanostructured MoS_3_/np-Mo catalysts improves the efficiency of the HER reaction, due to both the increased sulfur concentration in the amorphous molybdenum sulfide film and the synergistic effect of Mo nanoparticles on the catalytic properties of these films. With an increase in the loading of this catalyst, however, one can expect a significant decrease in the transparency of MoS_3_/np-Mo films due to reflection and scattering of light by metal nanoparticles. Therefore, it is necessary to develop a different technique for producing catalytic MoS*_x_* nano-layers with a controlled concentration of sulfur atoms. This technique can be reactive PLD (RPLD), which consists of pulsed laser ablation of a Mo target in H_2_S (hydrogen sulfide) gas [26]. When adjusting the composition of MoS*_x_* films, their optical and electronic properties can significantly change. Therefore, for a sufficiently complete understanding of the factors affecting the efficiency of photoelectrocatalytic HER processes, the energy band structures of these molybdenum sulfides should be studied, including in a heterostructure with a sublayer of a tungsten oxide semiconductor. To create a metal oxide layer in the MoS*_x_*/WO_3_ heterostructure, the RPLD method from the W target in dry air of varying pressure was used. RPLD modes were selected to provide the highest film growth rate and the formation of a fairly perfect WO_3_ nanocrystalline structure.

The energy band distribution for MoS*_x_*/WO_3_ heterostructures was studied by X-ray photoelectron spectroscopy (XPS) and photospectrometry (PS). It was revealed that, regardless of the chemical composition of the MoS*_x_* films, the photo-activated HER reaction on the surface of this structure developed according to the Z-scheme. The recombination of photogenerated electrons from the conduction band (CB) of WO_3_ and holes from the valence band (VB) of MoS*_x_* occurred at the film’s interface, which caused a decrease in the efficiency of electron–hole pair recombination in the bulk of catalytic MoS*_x_* film. The efficiency of the HER reaction depended substantially on the composition and chemical state of MoS*_x_* films. Both parameters can be effectively controlled by changing the H_2_S pressure during RPLD of these films.

It is known that the MoS_2_ compound is modified (oxidized) in an acid solution at potentials above 1.3 V [27]. Molybdenum oxysulfide is a more stable compound, and it has shown its promise as a material for cathodes and anodes. The energy band structures of molybdenum oxides and oxysulfides largely depend on their stoichiometry [28,29,30,31]. It was of interest to use porous MoS*_x_* nanofilms as precursors for porous MoO*_z_*(S) oxysulfides with various properties as well as to study their effect on the photoelectrocatalytic characteristics of nanostructured WO_3_ films in the OER reaction. MoO*_z_*(S) films formed during the anodic oxidation of MoS_3_ films turned out to be capable of effectively activating the oxygen evolution reaction at the WO_3_ photoanode.

This work shows that the layered heterostructure created by the RPLD technique on an FTO-coated glass substrate and containing MoS*_x_* and WO_3_ nanostructured films can be used to create semiconductor photocathodes and photoanodes (after electrochemical posttreatment), which ensure efficient splitting of water under solar light. Such a structure is formed through a relatively simple method in one chamber and using one technique. Laser ablation of the W target in an oxygen-containing gas is followed by laser ablation of the Mo target in hydrogen sulfide. Qualitative photocatalytic characteristics of the heterostructure are achieved by controlling the ablation modes of the targets and by implementing optimal conditions for the deposition of a laser plume in an O- and S-containing reaction medium.

## 2. Materials and Methods

### 2.1. Reactive Pulsed Laser Deposition and Posttreatment

Reactive pulsed laser deposition of WO*_y_* and MoS*_x_* films was carried out according to standard methodology. The laser radiation was directed at an angle of 45° to the target surface. The substrate for film deposition was installed normal to the direction of expansion of the laser erosion plume and parallel to the target surface. The substrate was 4 cm distanced from the target. The Solar LQ529 (Solar LS, Minsk, Belarus) laser was used for ablation of targets made of pure (99.99%) W and Mo metals. The laser radiation had the following characteristics: a wavelength of 1064 nm, a pulse duration of 15 ns, a pulse repetition rate of 20 Hz, a pulse energy of ~100 mJ, and a laser fluence of ~15 J/cm^2^ on the target. After W and Mo targets and substrates were installed in the chamber to obtain films, the chamber was pumped out by a turbomolecular pump to a pressure of ~10^−3^ Pa. Then, dry air was let into the chamber to a predetermined pressure, and the target W was ablated. Based on the results of previous studies by Fominski et al. [20], three dry air pressures of 20, 40, and 60 Pa were chosen to produce WO*_y_* films. The temperature of the substrate was 420 °C. The deposition time of tungsten oxide films was 20 min. To prepare a layered MoS*_x_*/WO*_y_*/substrate samples for photoelectrocatalytic water splitting, 0.5-mm thick FTO coated glass plates were used as substrates. The dimensions of the RPLD-covered area of the substrate used for photoelectrochemical investigation were 0.5 × 1 cm^2^.

After the RPLD process to obtain tungsten oxide films was completed, the chamber was again pumped out to a pressure of 10^−3^ Pa. The substrate with the deposited metal-oxide film was cooled to room temperature. Then, hydrogen sulfide was injected into the chamber to a predetermined pressure, and the Mo target was ablated. To obtain MoS*_x_* films with different sulfur contents, the H_2_S pressure was varied from 9 to 54 Pa. To measure the composition of MoS*_x_* films and their growth rate during RPLD, preliminary experiments were performed on the deposition of these films on polished Si substrates. The estimated deposition time on Si substrates was 2 min. When these films had been analyzed, the formation time of the catalytic nanolayer in the MoS*_x_*/WO_y_/FTO structure was determined. The time was chosen so that the formed MoS*_x_*/WO*_y_*/FTO samples contained the same amount of Mo atoms per cm^2^. For this, the RPLD time for producing MoS*_x_* films for MoS*_x_*/WO*_y_*/FTO test heterostructures increased from 4.5 to 9 min with a growth in the H_2_S pressure from 9 to 54 Pa.

To obtain MoO*_z_*(S)/WO*_y_*/FTO photoanodes, the prepared MoS*_x_*/WO*_y_*/FTO samples were oxidized in a 0.5 M H_2_SO_4_ solution with a potential of 2 V relative to the reversible hydrogen electrode (RHE). The time of the anode posttreatment ranged from 10 to 100 s.

### 2.2. Structural and Chemical Characterization Techniques

To determine the composition and thickness of thin MoS*_x_* films deposited on an Si substrate, as well as WO*_y_* films deposited on an FTO substrate, Rutherford backscattering spectroscopy (RBS) of helium ions was used. Research was conducted on an accelerator at the Immanuel Kant Baltic Federal University (Kaliningrad, Russia). The ion energy in the analyzing beam was 1.5 MeV. The chosen ion scattering angle was 160 °C. The measured RBS spectra of the ions were processed using SIMNRA software (SIMNRA 7.0, Garching, Germany).

The surface morphologies of the WO*_y_* and layered MoS*_x_*/WO_y_ films prepared on the FTO substrates were studied using scanning electron microscopy (SEM, Tescan LYRA 3, Brno, Czech Republic). FTO substrates with deposited MoS*_x_*/WO*_y_* films were split, and the fracture of the samples was studied with SEM. This procedure made it possible to examine the cross-sectional morphologies of the prepared films. The crystalline structure of WO*_y_* films deposited on the FTO substrate were investigated by grazing incidence X-ray diffraction (XRD, Cu Kα radiation, Ultima IV, Rigaku, Tokyo, Japan) and micro-Raman spectroscopy (MRS) using a 632.8-nm (He–Ne) laser. The cross-section of the laser beam was <1 μm. 

To reveal the structural features of MoS*_x_* films obtained by RPLD, thin MoS*_x_* films were separated from the Si substrate in an alkaline solution. Then, they were transferred onto metal grids and studied by transmission electron microscopy and selected area electron diffraction (TEM and SAED, respectively, JEM-2100, JEOL, Tokyo, Japan). The structural state of MoS*_x_* films was also studied by MRS using a 632.8-nm (He–Ne) laser. Such studies were performed for MoS*_x_* films deposited on Si substrates. The deposition time of these films for MRS studies was increased to 10 min. 

The chemical states of WO*_y_* and MoS*_x_* films were studied by XPS. XPS spectra were obtained by a Theta Probe Thermo Fisher Scientific spectrometer (Madison, WI, USA) with a monochromatic Al Kα X-ray source (*hν* = 1486.7 eV) and an X-ray spot size of 400 μm. The spectrometer energy scale was calibrated using Au 4f_7/2_ core level lines located at a binding energy of 84.0 eV.

### 2.3. Energy Band Structure Measurements

The band gaps (*E*_g_) for the WO*_y_*, MoS*_x_*, and MoO*_z_*(S) films were measured through the optical method by processing absorption spectra. To this end, a Tauc plot was constructed that described the dependence between (*αhν*)^1/*r*^ and (*hν*), where α is the absorption coefficient, *hν* is the photon energy, and *r* is a parameter that is taken to be 2 for indirect transitions. The optical absorption and transmission spectra were measured using an Agilent Technologies Cary Series UV–Vis–NIR spectrophotometer (Santa Clara, CA, USA).

XPS measurements were used to determine the mutual arrangement of valence bands in the created semiconductor heterostructures according to a technique that is widely used at present to study the band structure in heterojunctions [32,33,34]. This method is based on determining the positions of core levels relative to the edge of the valence band for each of the semiconductors individually and as part of the heterojunction. It was assumed that the position of the core level relative to the edge of the valence band did not change when the heterojunction was formed. Then, when it was possible to determine the shift between the core levels of semiconductors in the heterojunction, the valence band offset (VBO) could be calculated. To determine VBO in MoS*_x_*/WO*_y_* and MoO*_z_*(S)/WO*_y_* heterostructures on FTO substrates, the following measurements were performed. First, the XPS spectra (binding energies, *E*) of the Mo 3d and W 4f core levels were measured along with the spectra of the valence bands of sufficiently thick films (more than 3 nm) on FTO substrates. Then, the spectra of the Mo 3d and W 4f core levels were measured for MoS*_x_*/WO_3_ and MoO_z_(S)/WO_y_ heterostructures in which the thickness of the upper layer (MoS*_x_* and MoO*_z_*(S)) did not exceed 3 nm. Then, the VBO value for heterojunctions could be determined by the following formula:

*VBO* = (*E*_Mo3d5/2_ − *VBM*_Mo_) _bulk_ − (*E*_W4f7/2_ − *VBM*_W_)_bulk_ − (*E*_Mo3d5/2_ − *E*_W4f7/2_)_interface_,

where *VBM*_W_ and *VBM*_Mo_ are the energies of the upper edge of the valence band for WO_3_ and MoS*_x_* (or MoO_z_(S)), respectively. “Interface” stands for spectra of heterojunctions and “bulk” for spectra of thicker films on FTO substrates.

To determine the work function (*φ*), CutOff XPS spectra were measured. The *E*_k_^cutoff^ position was determined by extrapolating the tilt line to the zero baseline. The value of *φ* was determined by the formula *φ* = *hν* + *E*_k_^cutoff^ − *E*_F(polarized)_, where *hν* is the energy of the quanta of the exciting X-ray radiation and *E*_F(polarized)_ is the position of the Fermi level [35].

### 2.4. Photoelectrochemical Measurements of HER for MoS_x_/WO_y_ Heterostructures

To study the photoelectrocatalytical properties of MoS*_x_*/WO*_y_*/FTO samples in HER, these samples were illuminated by radiation of Xe lamps with a power of 100 W in a 0.5 M H_2_SO_4_ aqueous solution. The light intensity was maintained at 100 mW/cm^2^. A three-electrode configuration was used to measure the photo-activated current in an electric circuit with modified cathodes. The polarization curves were measured using linear sweep voltammetry (LSV) with a change of the applied potential from −400 to 0 mV and a scan rate of 2 mV/s. When measuring the LSV curves and the time evolution of the photocurrent, the light source was turned on and off. For chronoamperometry measurements, the potential of the tested samples was maintained at zero level (relative to RHE).

### 2.5. Preparation and Characterization of MoO_z_(S)/WO_y_ Photoanodes

After the MoO*_z_*(S)/WO*_y_*/FTO heterostructure had been formed by anodic posttreatment, the photoelectrocatalytic activity of these samples in the OER was studied. To do this, the polarization curves were measured with and without lighting, using linear sweep voltammetry (LSV) with an increase of the applied potential from 0 to 2 V (RHE) and a scan rate of 2 mV/s. The optical properties, chemical states, and energy bands of MoO*_z_*(S)/WO*_y_*/FTO samples were studied, which made it possible to determine the mechanism of influence of MoO*_z_*(S)films on the photoelectrocatalytic activity of WO*_y_* films in the OER process. 

## 3. Results

### 3.1. The Structure and Composition of WO_y_ Films

**Figure 1:**
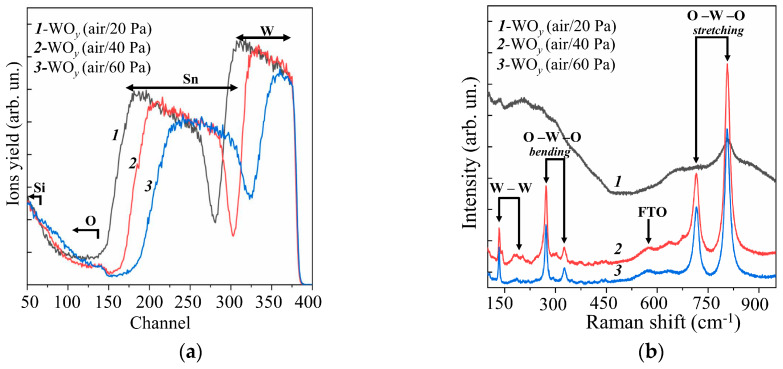
Experimental (**a**) RBS and (**b**) Raman spectra for the WO*_y_* films obtained on an FTO substrate by RPLD at different pressures of dry air.

Figure 1a shows RBS spectra for WO*_y_* films obtained by RPLD on FTO substrates at various pressures of dry air. The mathematical processing of these spectra using the SIMNRA software showed that an increase in dry air pressure from 20 to 60 Pa caused a decrease in the deposition rate of W atoms by about a factor of two (see Supplementary Materials, Figure S1). The estimated thickness of the WO*_y_* films decreased from ~350 to ~150 nm. Thus, the WO*_y_* film thickness (~250 nm) obtained by RPLD at dry air pressure of 40 Pa in this work was approximately equal to the thickness of the amorphous WO*_y_* film, which was obtained by RPLD previously [20]. This made it possible to reveal the effect of the structural state of the metal oxide films on the photoelectrocatalytic HER performance of MoS*_x_*/WO*_y_* heterostructures. For all the obtained WO*_y_* films, the ratio *y* = O/W exceeded 3. This could be due to the fact that oxygen and water molecules from the ambient air could penetrate into the pores of WO*_y_* films and remain there even when the samples were placed in a vacuum chamber for RBS studies. These results indicated a relatively low density of the formed WO*_y_* films. 

MRS studies of WO_y_ films showed (Figure 1b) that a fairly perfect crystalline structure of WO*_y_* films was formed at dry air pressures of 40 and 60 Pa. This was indicated by the peaks characteristic of the WO_3_ monoclinic phase at 271.8, 722.3, and 806.7 cm^−1^ [36,37]. In this case, no obvious differences in the MRS spectra of these films were observed. At lower dry air pressure (20 Pa), the Raman spectrum of the WO*_y_* film consisted of several broadened bands; this pointed to a strongly disordered/amorphous structure of the film. Based on the results of RBS and MRS studies, it was decided to use WO*_y_* films obtained at an air pressure of 40 Pa to form photocatalytic heterostructures. At that dry air pressure, the formation of sufficiently perfect crystalline films was observed under the condition of the highest rate of their deposition by RPLD.

**Figure 2:**
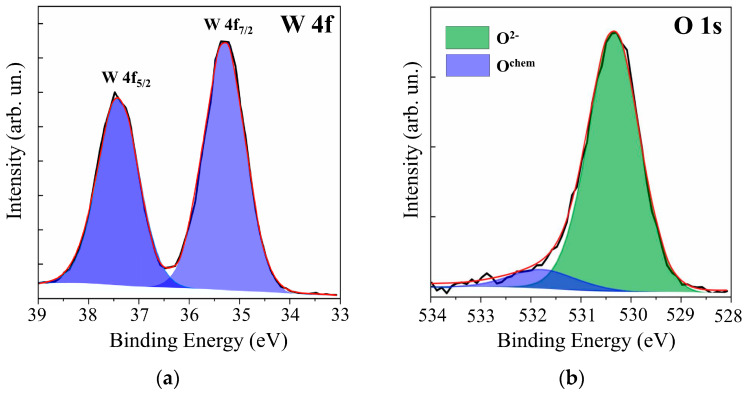
XPS (**a**) W 4f and (**b**) O 1s spectra for the surface of WO*_y_* film, which was obtained by RPLD at a dry air pressure of 40 Pa.

Additional studies of WO*_y_* films by XPS showed (see Figure 2) that, in the case of RPLD at a dry air pressure of 40 Pa, quite effective oxidation of W atoms occurred. The XPS spectrum analysis of W 4f revealed the dominance of W^6+^ states with binding energies for W 4f_7/2_ − 4f_5/2_ peaks equal to 35.3 and 37.4 eV. The O 1s peak corresponding to O‒W states was located at a binding energy of 530.4 eV. The peak at 531.8 eV arose from O-containing surface contaminants. These parameters of XPS spectra for WO*_y_* films indicated the formation of a fairly perfect WO_3_ compound [37,38,39]. The calculation of *y* = O/Mo according to XPS showed that *y* ≈ 3. In view of the results of structural studies, WO*_y_* films obtained at a dry air pressure of 40 Pa were designated as WO_3_. In the analysis of XPS data for WO_3_ films, the possibility of the formation of W^5+^ (BE W 4f_7/2_ ~35.2 eV, BE O 1s ~531 eV) was taken into account. There was, however, no reliable confirmation that a noticeable concentration of oxygen vacancies had formed in the surface layer of these films.

**Figure 3:**
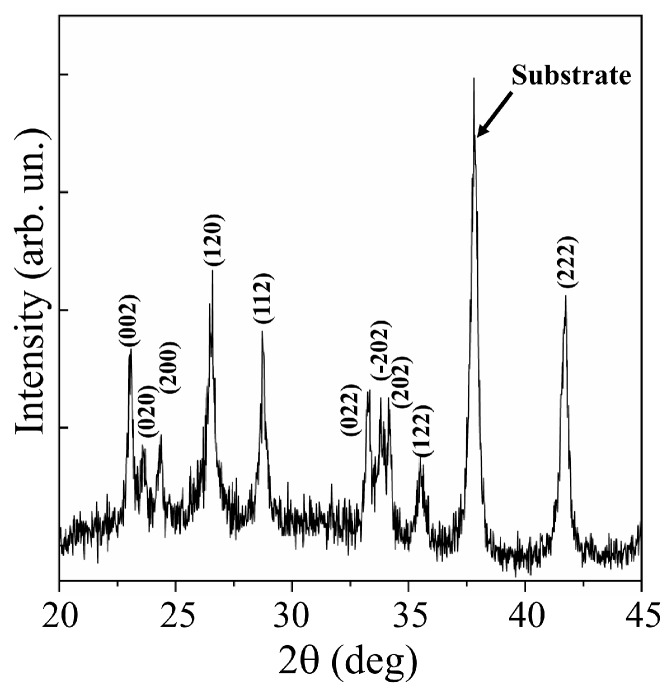
XRD pattern for WO_3_ film obtained on an FTO substrate by RPLD at a dry air pressure of 40 Pa.

XRD studies of WO_3_ films obtained at an air pressure of 40 Pa confirmed a fairly perfect crystalline structure of these films (Figure 3). Analysis of the XRD pattern for the WO_3_/FTO sample showed that sets of peaks corresponding to the WO_3_ phase with the P21/*n* (14) monoclinic lattice (PDF # 01-071-2141) were suitable for experimental peaks. In the X-ray diffraction pattern for this film, there were no peaks that would stand out in intensity among other peaks. This indicated that the film had grown in a polycrystalline mode.

Mai et al. [38] performed reactive pulsed deposition of WO*_y_* films in oxygen at 400 °C. An excimer laser was used to ablate the WO_3_ target. In X-ray diffraction patterns of WO_3_ films obtained on FTO substrates at relatively high oxygen pressures (≥13 Pa), intensity peaks at 23.6°, 24.2°, and 24.8° corresponding to the (002), (020), and (200) planes, respectively, were distinguished. The authors attributed the appearance of these peaks to the formation of a monoclinic WO_3_ phase, the texture of which depended substantially on the oxygen pressure. In the X-ray diffraction pattern of the WO_3_ film obtained using a pulsed Nd:YAG laser (Figure 3), the peaks for these planes were located at 23.1°, 23.6°, and 24.4°. The difference in lattice parameters for WO_3_ films obtained using solid-state and excimer lasers could be due to a combination of factors affecting film growth. Such factors include the film deposition rate (the mass of ablated material per pulse), as well as the composition and pressure of the reaction medium. The position of the peaks in the XRD pattern for the WO_3_ film obtained by RPLD with a solid-state laser coincided quite well with the positions of the peaks from the WO_3_ monoclinic phase, which was formed in WO_3_ films using more traditional/chemical methods for synthesizing this metal oxide (for example, [37]). 

**Figure 4:**
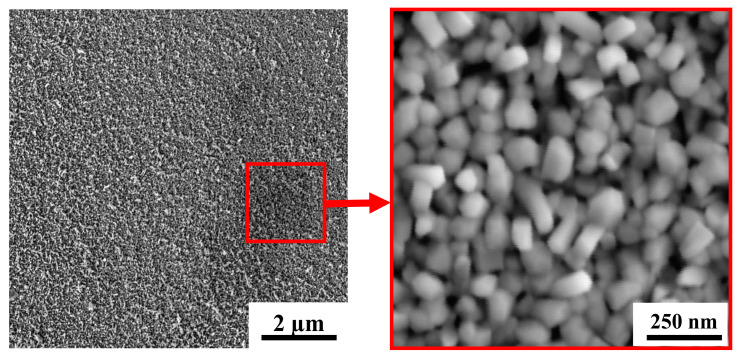
SEM images with two magnifications for a WO_3_ film obtained on an FTO substrate by RPLD at a dry air pressure of 40 Pa.

Figure 4 shows SEM images for a WO_3_ film deposited on an FTO substrate by RPLD at a dry air pressure of 40 Pa. The films obtained under these conditions consisted of nanocrystals in the form of up to 100-nm long nanorods. The packing density of WO_3_ nanocrystals was low, and they were oriented randomly relative to the film surface. Comparison of this structure with the structure of WO*_y_*_<3_ films, which were obtained by Fominski et al. [20] using PLD at room temperature for FTO substrates, showed that deposition on a heated FTO substrate changed the growth mechanism of WO*_y_* films and contributed to an increase in their porosity. Deposition on a heated FTO substrate disrupted the substrate-to-surface film growth and caused non-aligned growth of nanocrystals. A similar growth mechanism was found upon the deposition of WO*_y_* films on heated single-crystal SiC substrates [40]. Variation of RPLD conditions on FTO substrates did not make it possible to grow a highly porous WO*_y_* film consisting of needle nanocrystals. At all the pressures studied, films of nanorods oriented randomly relative to the surface were formed (Figure 4 and Supplementary Materials Figure S2). Previously, the formation of nanocrystals in the form of thin needles oriented perpendicular to the surface of the substrate was detected during the RPLD of WO*_y_* films on heated glassy carbon substrates [41].

**Figure 5:**
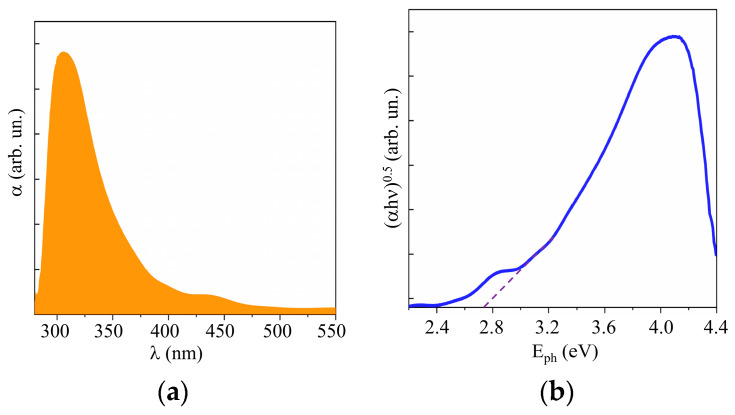
(**a**) UV–visible absorption spectra and (**b**) Tauc plot of a WO_3_ film obtained on FTO substrate by RPLD at a dry air pressure of 40 Pa.

Studies of the optical properties of WO_3_ films obtained by RPLD showed that these properties were largely comparable with the properties of WO_3_ films obtained by other synthesis methods [42,43]. The UV–visible absorption spectra of the RPLD WO_3_ film showed the absorption onset of ~450 nm, as shown in Figure 5a. The band gap was *E*_g_ ~ 2.75 eV (Figure 5b). WO_3_ films with a perfect crystal structure had very weak absorption in the visible region [42]. The absorption spectra of WO_3_ obtained by RPLD were characterized by the tailing of absorbance onset into the visible region; this indicates the presence of oxygen vacancies in the bulk of these films. The peak at 450–500 nm in the spectrum could be caused by an interference effect in multilayer films [38]. An XPS study of the valence band showed that the Fermi level was more than 2 eV away from the upper edge of the valence band (see Supplementary Materials Figure S3). The WO_3_ films thus had n-type conductivity due to the presence of vacancies in the crystal lattice. 

### 3.2. The Structure and Composition of MoS*_x_* Films

**Figure 6:**
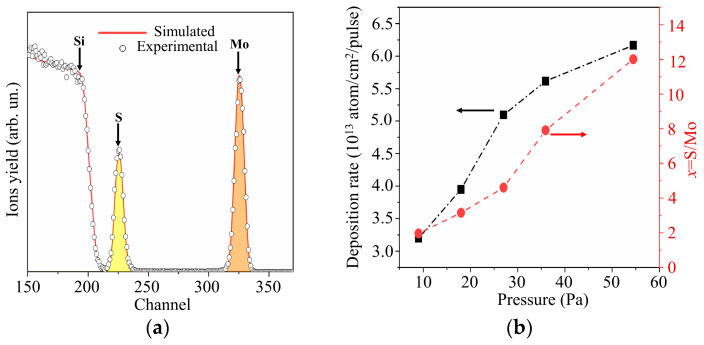
(**a**) The RBS spectrum for MoS*_x_* film obtained by RPLD on a Si substrate at an H_2_S pressure of 27 Pa; (**b**) deposition rates and compositions of MoS*_x_* films obtained by RPLD at different pressures of H_2_S gas.

Figure 6a shows the RBS spectrum characteristic of a MoS*_x_* thin film obtained by RPLD on a polished Si substrate at an H_2_S pressure of 27 Pa. The experimental spectrum was in good agreement with the model spectrum, which corresponded to a MoS*_x_* film on silicon at *x* ~ 4.5. Figure 6b shows the results of an RBS study of MoS*_x_* films produced by RPLD at various H_2_S pressures. An increase in the pressure of hydrogen sulfide from 9 to 54 Pa caused an increase in the sulfur content in MoS*_x_* films. The efficiency of introducing sulfur into the films turned out to be so high that, with an increasing pressure of hydrogen sulfide, the overall deposition rate rose despite a decrease in the deposition rate of Mo atoms. The reduction in the deposition rate of Mo atoms with increasing background gas pressure is explained by the scattering of the laser plume on gas molecules [44].

Taking into account the results of RBS measurements of the sulfur content in MoS*_x_* films shown in Figure 6b, the films were designated as follows: MoS_2_ (pressure H_2_S was 9 Pa), MoS_3.2_ (18 Pa), MoS_4.5_ (27 Pa), MoS_8_ (36 Pa), and MoS_12_ (54 Pa). An XPS study of these films showed that, overall, the RBS and XPS measurements of sulfur content correlated well with each other. The *x* values measured by the XPS exceeded, however, by 10%–15% those measured by RBS. This could be due to the fact that, after the termination of RPLD, H_2_S molecules could be adsorbed on the surface of all MoS*_x_* films, and additional Mo‒S states could be formed. The efficiency of this process could not be high, since hydrogen sulfide did not show high chemical activity in the absence of laser plasma. The effect of this process on the chemical composition of the surface of MoS*_x_* films still cannot be ruled out.

**Figure 7:**
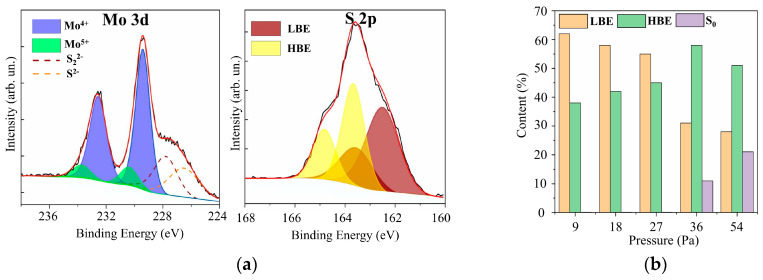
(**a**) The XPS Mo 3d and S 2p spectra for a MoS_4.5_ film obtained by RPLD at an H_2_S pressure of 27 Pa; (**b**) the relative content of low binding energy (LBE), high binding energy (HBE), and S_0_ states of sulfur atoms in MoS*_x_* films obtained by RPLD at different pressures of H_2_S gas.

In the XPS spectra of Mo 3d for films with different sulfur contents (see Figure 7 and Supplementary Materials Figure S4), two doublets Mo 3d_5/2_–3d_3/2_ corresponding to Mo^4+^ and Mo^5+^, could be distinguished. The Mo^4+^ state, which corresponded to the Mo 3d_5/2_ and 3d_3/2_ peaks at 229.4 eV and 232.5 eV, respectively, is formed in nanomaterials with a local atom packing characteristic of the 2H–MoS_2_ phase and in amorphous compounds containing 3Mo-S clusters [45,46,47]. In 3Mo-S clusters, three Mo atoms are connected in a triangle and surrounded by sulfur ligands, the features of which are manifested in S 2p spectra. The Mo^5+^ state is more characteristic of amorphous MoS*_x_* compounds containing Mo–S3 clusters [48,49]. In the local regions of these nanomaterials, Mo atoms are connected in a linear chain through three S atoms. For the RPLD MoS_2_ film, the spectrum contained a third doublet with Mo 3d_5/2_–3d_3/2_ peaks at 232.1 and 235.2 eV; this doublet corresponded to Mo^6+^ in compounds with oxygen. Mo–O states could form on the surface of MoS*_x_* samples after they were removed from the vacuum chamber to air [20]. With an average value of *x* ~ 2, on the surface of the film, there may have been local sections of sub-stoichiometric composition that interacted effectively with oxygen.

The XPS S 2p spectra analysis showed (Figure 7 and Supplementary Materials Figure S4) that these spectra contained two doublets that are usually characterized as those with low and high binding energies [45,46,47]. The S 2p_3/2_–2p_1/2_ doublet with a low binding energy (162.3 and 163.5 eV for S 2p_3/2_ and S 2p_1/2_ peaks, respectively) is characteristic of the states of S^2−^ atoms in the crystalline MoS_2_ or unsaturated S^2−^ in the amorphous MoS*_x_* [45,46,47]. The low-binding doublet may also correspond to terminal S_2_^2−^ in the amorphous MoS*_x_* and 3Mo-S clusters. The presence of a doublet with a high binding energy, in which S 2p_3/2_ and S 2p_1/2_ peaks had a binding energy of 163.1 and 165.0 eV, respectively, indicated the possibility of the formation of the following sulfur ligands in amorphous or clustered-type (Mo–3S and 3Mo–S) films: bridging S_2_^2−^, shared S_2_^2−^, and/or apical S^2−^ [45,50,51,52].

The RPLD process at pressures of more than 36 Pa caused an increase in the sulfur concentration in MoS*_x_* films; this led, however, to the appearance of polysulfide inclusions (S_0_) in these films. In the XPS spectra (Supplementary Materials Figure S3) of the films obtained at a pressure of 36 Pa, a third doublet appeared, in which the S 2p_3/2_ peak was located near the binding energy of 164 eV. With an increase in pressure to 54 Pa, the relative intensity of this doublet increased even more. The formation of polysulfide clusters proceeds mainly under conditions of reduced chemical activity of the deposited components of MoS*_x_* films. For example, S_0_ polysulfide clusters were detected in MoS*_x_* films obtained by pulsed laser ablation of the MoS_2_ target under conditions of increased buffer gas pressure [53]. This gas could lower the energy and chemical activity of the deposited Mo and S atoms significantly; the interaction of sulfur atoms with each other became more effective than the interaction of sulfur with molybdenum. Figure 7b shows the effect of hydrogen sulfide pressure on the distribution of sulfur between the three main chemical states in MoS*_x_* films. The increase in H_2_S pressure caused an increase in the proportion of states characterized by a high binding energy. At pressures above 36 Pa, however, a relatively sharp decrease in the states with a low binding energy was observed, and S_0_ states were formed instead.

**Figure 8:**
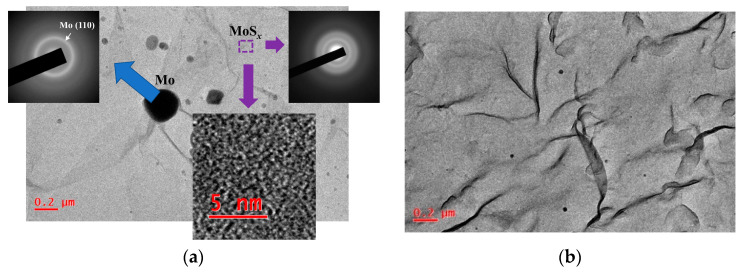
(**a**) TEM image and selected area diffraction (SAED) patterns of the thin MoS_4.5_ film obtained by RPLD (the local area of the film that contained Mo particles was selected). The bottom insert shows high-resolution TEM image of the MoS_4.5_ film. (**b**) Typical TEM image of the MoS_4.5_ film, which indicates a low content of Mo particles embedded in this film.

TEM/SAED studies of MoS*_x_* thin films showed that the films had an amorphous structure (Figure 8). The contrast of high-resolution TEM images consisted of randomly oriented threadlike fragments of dark and light shades. For all the MoS*_x_* films, several diffusely broadened rings were present on SAED patterns. The two most intense rings corresponded to wave vectors with modules equal to ~24 and ~35 nm^−1^, and they were caused by the electron diffraction in MoS*_x_* clusters. These films contained nanosized Mo particles that were ejected from the Mo target during pulsed laser ablation. The largest particle size was ~200 nm (Figure 8a). However, the concentration of such relatively large particles on the surface of the MoS*_x_* film was low. Mostly the particles smaller than 20 nm in diameter predominated (Figure 8b). The average concentration of such particles on the film surface did not exceed 10 particles per 100 μm^2^. These particles would not significantly affect the photoelectrochemical performance of the prepared heterostructures. It should be noted that when choosing the conditions for laser ablation of the Mo target, we tried to reduce the influence of this process. However, complete suppression of the laser-induced particle ejection from the target was difficult. The mechanisms of phase explosion and/or hydrodynamic liquid phase spraying may cause the formation of particles when intensities of the pulsed laser ablation are varied from higher to relatively low [54,55,56].

**Figure 9:**
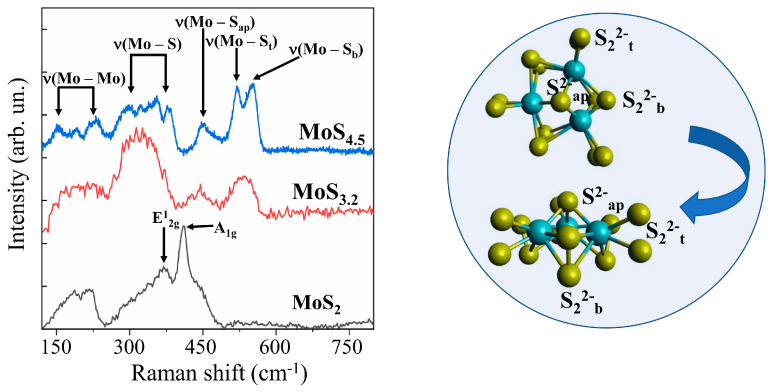
Raman spectra for different MoS*_x_* films obtained by RPLD. The insert shows terminal (S_2_^2−^_t_), bridging (S_2_^2−^_b_), and apical (S^2−^_ap_) ligands in a 3Mo‒S (Mo_3_S_13_) cluster.

Micro-Raman spectroscopy turned out to be a more informative method for identifying the local structure features of MoS*_x_* films (Figure 9). To obtain sufficiently intense MRS spectra, thicker MoS*_x_* films were produced by RPLD. By the type of MRS spectra, all the films could be divided into three groups. In addition to the broadened bands, the MRS spectra of MoS_2_ films included narrow peaks at 370 and 410 cm^−1^. These peaks were probably due to the vibrations of A_1g_ and E_2g_^1^ in nanoclusters with a laminar atom packing characteristic of the 2H–MoS_2_ phase [57]. Only a few broad peaks at 200, 320, 450, and 540 cm^−1^ were present in the Raman spectra of the MoS_3.2_ films. Such a spectrum is characteristic of amorphous nanomaterials in which there is no sufficiently perfect atomic ordering into 3Mo-S clusters [46,58]. In films with a high sulfur content (MoS_4.5_, MoS_8_, and MoS_12_), the local ordering of atomic packing into clusters of this type led to the appearance of narrow peaks attributed to the following vibration modes: *ν*(Mo–Mo) at ~200 cm^−1^, *ν*(Mo–S)_coupled_ at ~320 cm^−1^, *ν*(Mo–S_apical_) at ~450 cm^−1^, *ν*(S–S)_terminal_ at ~520 cm^−1^, and *ν*(S–S)_bridging_ at 540 cm^−1^.

**Figure 10:**
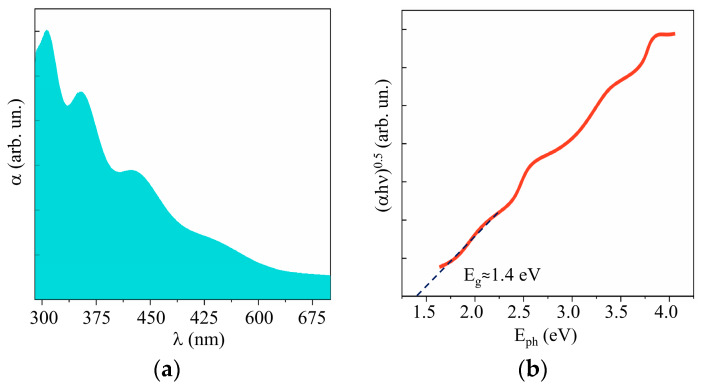
(**a**) UV–visible absorption spectra and (**b**) Tauc plot of MoS_4.5_ film.

Figure 10 and Supplementary Materials Figure S5 show the results of a study into the optical properties of MoS*_x_* films. These films absorbed visible light more efficiently than WO_3_ films did. The band gap depended weakly on the sulfur concentration and was ~1.6, 1.5, and 1.4 eV, respectively, for the MoS_2_, MoS_3_._2_, and MoS_4.5_ films. According to the XPS studies of the valence band in quite thick MoS*_x_* films, the position of the Fermi level in the band gap depended on the sulfur concentration (see Supplementary Materials Figure S6). However, for all the films, the Fermi level was shifted closer to the valence band, which pointed to p-type conductivity mechanism in these films. 

**Figure 11:**
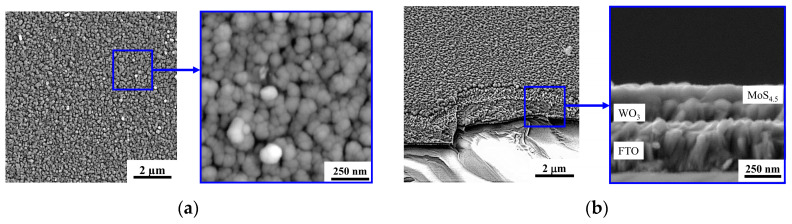
The (**a**) planar and (**b**) cross-section SEM images of the MoS_4.5_/WO_3_/FTO sample.

Reactive PLD of the MoS*_x_* films on the porous WO_3_ film led to the formation of a porous bilayer film, the characteristic SEM image of which is shown in Figure 11. Morphology of the “cauliflower” type was typical of MoS*_x_* films deposited by RPLD on glassy carbon [26]. The same morphology with a large catalytically active surface was also formed during RPLD of MoS*_x_* films on porous WO_3_. A separate “bush” of the MoS*_x_* film nucleated on the surface of the WO_3_ film, and during growth, the transverse size of the bush increased in size, reaching ~70 nm.

### 3.3. Electrochemical Posttreatment of MoS_x_ Films

**Figure 12:**
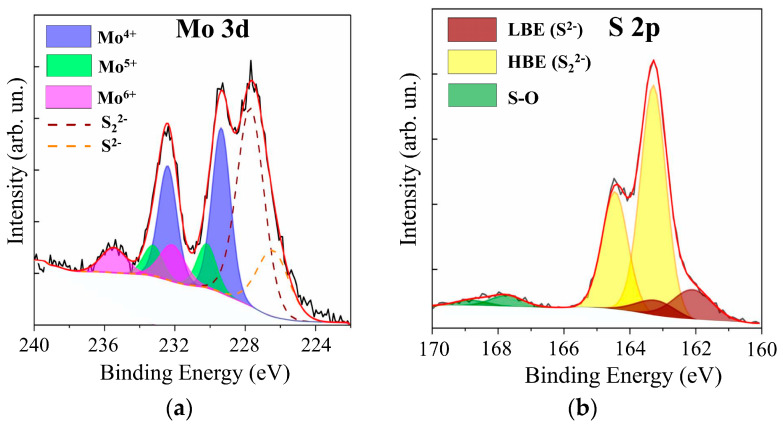
The XPS (**a**) Mo 3d and (**b**) S 2p spectra for a MoS_3.2_ film obtained by RPLD at an H_2_S pressure of 18 Pa and post-treated in acidic solution at a potential of 2 V.

Figure 12 shows XPS data for the MoO*_z_*(S) film, which was obtained by anodic posttreatment of a MoS_3.2_ film. The anode posttreatment time at a potential of 2 V was 100 s. Comparison of this data with XPS data for the as-prepared MoS_3.2_ film (see Supplementary Materials Figure S4) showed that posttreatment caused a noticeable decrease in the molybdenum content in these films. The S 2s peak was equal in intensity to the Mo 3d peak. Moreover, in the XPS S 2p spectrum, the relative intensity of the doublet with a low binding energy attributed to the S^2−^ states decreased, whereas the intensity of the doublet corresponding to the S_2_^2−^ states increased. XPS measurement of the film composition showed that, prior to treatment, the composition was described by the formula MoS_3.3_, and after the treatment, the composition was described by MoO_3_S_8.5_.

Posttreatment led to the incorporation of oxygen into the film and the formation of Mo‒O, Mo‒O‒S, and S‒O chemical bonds [30,31,59,60,61]. In the XPS Mo 3d spectrum, this was indicated by the appearance of the Mo 3d_5/2_–3d_3/2_ doublet with peak binding energies of 232.2 and 235.5 eV, respectively. This doublet pointed to the appearance of Mo^6+^ oxidation states. A doublet was detected in the XPS S 2p spectrum, in which S 2p_3/2_ and S 2p_1/2_ peaks had binding energy of 167.8 and 168.5 eV, respectively. The posttreatment of MoS_3.2_ films caused an increase in the contribution of the doublet that corresponded to the Mo^5+^ oxidation state to the XPS Mo 3d spectrum. The ratio of Mo^5+^/Mo^4+^ increased from 0.14 to 0.27. This could be due to the fact that the anodic oxidation of the MoS*_x_* film caused the formation of the Mo‒O‒S ternary compound, in which Mo^5+^ oxidation states were realized [60]. 

The studies of the main characteristics of the energy band structures of MoO*_z_*(S) films showed that the band gap was ~1.55 eV (see Supplementary Materials Figure S7a), whereas the Fermi level was 1.1 eV away from the bottom of the energy band gap for thin MoO*_z_*(S) films (see Supplementary Materials Figure S7b). Comparison of the characteristics of MoO*_z_*(S) films with the characteristics of MoS*_x_* precursor films showed that the oxidation of the MoS*_x_* film caused a slight increase in the band gap. The optical properties of MoO*_z_*(S) films can depend substantially on the chemical composition these films [30,61]. With an increase in the oxygen concentration in the ternary compound, however, an increase in the band gap should be expected, since the physical properties of this compound approach the properties of MoO_3_, whose band gap is ≥3 eV [60,61,62,63,64]. A shift in the Fermi level to the upper edge of the band gap indicated that the formation of Mo^6+^‒O and Mo^5+^‒O‒S bonds in MoO*_z_*(S) films could cause a change in the type of conductivity of these films due to an increase in the concentration of donor states. It is known that MoO_3_ films have an *n*-type conductive mechanism if their chemical composition is close to stoichiometric [64].

### 3.4. The Photoelectrocatalytical HER Performance of MoS_x_/WO_3_/FTO Samples

**Figure 13:**
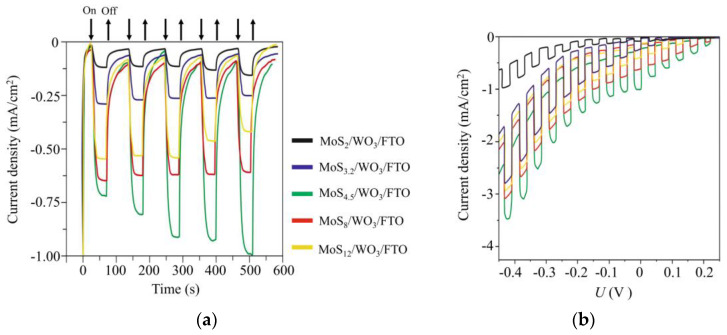
(**a**) Chopped (light/dark) chronoamperometry curves and (**b**) chopped linear sweep voltammetry (LSV) curves of MoS*_x_*/WO_3_/FTO photocathodes with a different stoichiometry of MoS*_x_* catalytic films. The curves were measured in a 0.5 H_2_SO_4_ solution at an applied potential of ~0 V (RHE) under Xe lamp illumination (~100 mW/cm^2^).

Figure 13 shows the chronoamperometry curves measured at an applied potential of ~0 V (RHE) under illumination, using chopped light for differently prepared MoS*_x_*/WO_3_/FTO samples. The highest photoelectrocatalytic activity was observed for the MoS_4.5_/WO_3_/FTO sample that contained a MoS_4.5_ film obtained by RPLD at an H_2_S pressure of 27 Pa. For this sample, the transient photocurrent response increased over time and reached ~1 mA/cm^2^ under illumination. This photocurrent value was maintained during measurements, the duration of which reached several days (Supplementary Materials Figure S8).

The thicknesses of the WO_3_ and MoS_4.5_ layers were ~250 and ~100 nm, respectively. It should be noted that we did not perform accurate optimization of the thickness of the MoS*_x_* films. Previously, we found that the deposition of thinner MoS*_x_* films caused a deterioration in photoelectrocatalytic HER performance of MoS*_x_*/WO_3_/FTO samples, and thicker MoS*_x_* films had poor transparency. The loading of the MoS*_x_* catalytic film was chosen so that MoS*_x_*/WO_3_/FTO samples absorbed only part of the photon flux (see Supplementary Materials Figure S9). Having passed through the photocathode, the light beam may be used to photoactivate the electrochemical process of OER on a photoanode, e.g., MoO_z_(S)/WO_3_/FTO, if it will have an appropriate optical property. To this end, the photoanode will be installed parallel to the photocathode and illuminated by the light passing through the photocathode. 

An analysis of the efficiency of photoactivated HER dependence on the composition of MoS*_x_* films showed that this efficiency increased significantly with increasing sulfur concentration from *x* ~ 2 to *x* ~ 4.5. Then, the effectiveness of photoactivated HER decreased as sulfur concentration increased. RBS measurements showed that an increase in the concentration of sulfur in MoS*_x_* films was accompanied by an increase in its total content in the films. The growth rate of the photocurrent was, however, more substantial than the growth rate of the total amount of sulfur in the films. This could be due to the fact that, with increasing sulfur concentration, the proportion of sulfur atoms that were characterized by a high binding energy in the XPS spectra increased (Figure 7b). The increased catalytic activity of such sulfur atoms (bridging S_2_^2−^ and/or apical S^2−^) in the HER reaction is noted in [45]. A series of studies revealed the transformation of the structure and composition of amorphous MoS*_x_*_≥3_ catalysts during cathodic pretreatment [46,47,50]. It is assumed that the composition and states characteristic of MoS_2_ are formed in the surface layer of these catalysts. Excess sulfur is removed from the surface of MoS*_x_*_≥3_ catalysts in the form of H_2_S molecules. Obviously, with such a mechanism of the HER process, the surface density of catalytically active states should correlate with the content of Mo atoms in MoS*_x_*_≥3_ films. In our studies, for MoS*_x_*_≥2_ films, the total Mo content was approximately the same. The photoelectrocatalytic activity of MoS*_x_*/WO_3_/FTO samples increased, however, with growing sulfur content, until polysulfide clusters appeared in the films. Figure 13 also shows that the photocurrent increased with time for the MoS_4.5_/WO_3_/FTO sample but decreased for the films possessing larger S contents. This means that when the Mo content remains largely unchanged, the amount of sulfur affects stability and catalytic activity.

### 3.5. The Photoelectrocatalytical OER Performance of MoO_z_(S)/WO_3_/FTO Samples

**Figure 14:**
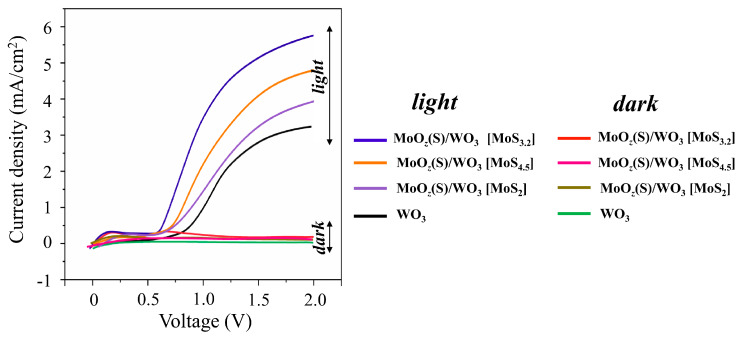
The LSV curves of WO_3_/FTO and different MoO*_z_*(S)/WO_3_/FTO samples measured in the dark and under illumination by a Xe lamp in 0.5 M H_2_SO_4._ The MoO*_z_*(S) films were prepared by posttreatment of MoS*_x_* precursors with different content of S atoms (indicated in square brackets).

Figure 14 shows LSV scans that were measured for WO_3_/FTO and different MoO_z_(S)/WO_3_/FTO samples without and under illumination by a Xe lamp. LSV curves for the WO_3_/FTO sample had a form characteristic of WO_3_-based photoanodes [38,39,42]. The photocurrent onset potential of the WO_3_/ FTO sample began at 0.8 V, and the photocurrent density increased steeply up to a plateau of about 2.9 mA/cm^2^ at 1.6 V. The received photocurrent for the WO_3_/FTO sample obtained by RPLD exceeded the photocurrent for WO_3_ films obtained by PLD by Mai et al. [38]. Comparison of WO_3_/FTO photoanodes obtained by RPLD in this work with WO_3_-based photoanodes obtained by various methods (see a review in [65]) indicates a rather high photoelectrochemical performance of WO_3_/FTO samples in O_2_ production by water splitting.

The formation of MoO*_z_*(S) films caused the modification of the OER performance of the WO_3_-containing photoanodes. The MoO*_z_*(S) films that were prepared by posttreatment of MoS_3.2_ precursors caused an almost twofold increase in the photocurrent (up to 5.3 mA/cm^2^ at 1.6 V) for the MoO*_z_*(S)/WO_3_/FTO sample compared to the photocurrent of the WO_3_/FTO sample. At the same time, the photocurrent onset potential decreased by about 0.2 V. The MoO*_z_*(S)/WO_3_/FTO photoanodes on the base of other MoS*_x_* precursors demonstrated the worse photoelectrochemical OER performances (Figure 14). The stability of the WO_3_/FTO and MoO*_z_*(S)/WO_3_/FTO photoanodes was measured under continuous illumination by a Xe lamp at a constant bias potential of 1.6 V (Supplementary Materials Figure S10). These measurements indicated that the MoO*_z_*(S) film did not cause a significant decrease in the stability of the photoanode during water splitting. After a fast drop in photocurrent at the beginning, the photocurrent slowly decreased for both photoanodes. The photocurrent remained ~70%–80% after 2 h illumination; this is typical of WO_3_-based photoanodes, as reported in [38,42]. 

## 4. Discussion

**Figure 15:**
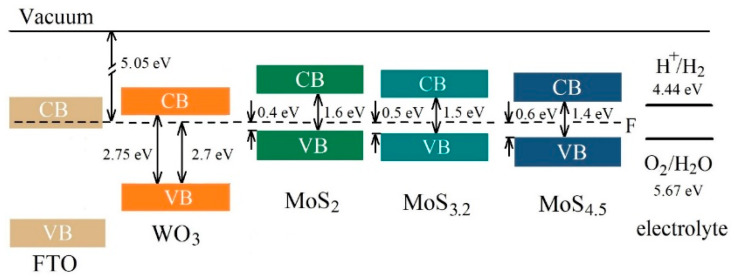
The proposed energy band diagrams for MoS_2_/WO_3_/FTO, MoS_3.2_/WO_3_/FTO, and MoS_4.5_/WO_3_/FTO samples. The schema represents the band offsets between WO_3_ with thin MoS*_x_* films. CB and VB indicate the conduction band and valence band, respectively.

Comparison of the results obtained in this work with the findings of Fominski et al. [20] showed that regulating MoS*_x_* catalyst loading and improving the structural and chemical state of catalytic MoS*_x_* and photoactive WO_3_ layers helped to increase the efficiency of photoelectrochemical HER nearly tenfold when using a MoS_4.5_/WO_3_/FTO sample. As mentioned above, the chemical composition of the MoS*_x_* film was able to affect the efficiency of the photoelectrochemical process of HER. This happened when the chemical composition of a MoS*_x_* film had little effect on the structure of the energy bands of the latter. Figure 15 shows the assumed band structure of MoS*_x_*/WO_3_/FTO samples with different MoS*_x_* films. The position of energy bands in FTO was determined according to [66]. The MoS*_x_* films were selected that had a significant dependence between the efficiency of the photoelectrochemical HER process in MoS*_x_*/WO_3_/FTO samples, on the one hand, and the sulfur concentration, on the other. The results of VB-XPS studies for MoS*_x_* and WO_3_ thin films were used to determine the energy band structures. These results take into account the interactions between films (the built-in charge) on the interface. When removed from the interface, the upper edge of the valence band could move to the Fermi level in thicker MoS*_x_* films (see Supplementary Materials Figure S6).

The parameters of the band structures indicate that an increase in the sulfur concentration in MoS*_x_* films, from *x* ~ 2 to *x* ~ 4.5, could not cause significant changes in the mechanism of the photoelectrochemical process of HER. In all MoS*_x_*/WO_3_ heterostructures, this process followed the Z-scheme mechanism of charge separation and transfer (see Supplementary Materials Figure S11). Light absorption caused the generation of electron‒hole pairs in MoS*_x_* and WO_3_ films. The mutual position of energy bands in those films is such that the photogenerated electrons with strong reduction ability in the CB of MoS*_x_* film are preserved since the photogenerated holes in the VB of MoS*_x_* film and photogenerated electrons in the CB of WO_3_ with inferior redox power recombine. The redox power of the charge carriers are determined by the position of CB and VB energy levels relative to the HER and OER potentials, which are indicated in Figure 15. Photogenerated holes in the valence band of WO_3_ probably recombine with electrons (from the conduction band of FTO), which are injected from an external circuit. Due to such a direct Z-schema charge carrier transfer pathway, the electron lifetime may be increased in a MoS*_x_* catalyst film, and the flux of electrons with strong reduction ability to the MoS*_x_*/electrolyte interface will grow as a result. Although MoS_2_, MoS_3.2_, and MoS_4.5_ films did not differ in the type of conductivity, a lower concentration of equilibrium holes in MoS_4.5_ films was able to affect (i.e., reduce) the recombination rate of photogenerated electrons and holes in those films and thus to facilitate an increase in the electron flux to the surface of a MoS_4.5_ catalyst film. The concentration of photogenerated carriers in the MoS_4.5_ film may be the highest because the energy band gap of MoS_4.5_ is minimal in comparison with that of the MoS_2_ and MoS_3.2_ films (Figure 15). Even though the demonstrated photo-activated HER performance of RPLD MoS*_x_*/WO*_y_* heterostructures is lower compared to some previous studies of heterostructures that were prepared by PLD (e.g., CuBi_2_O_4_/NiO [67]), the thickness of the functional layers of obtained MoS*_x_*/WO*_y_* samples could be optimized to enhance their photoelectrocatalytic characteristics. 

**Figure 16:**
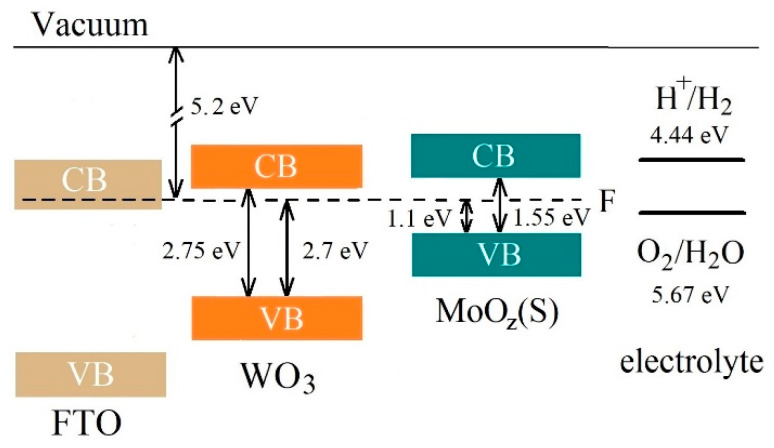
The proposed energy band diagram for MoO*_z_*(S)/WO_3_/FTO sample. The schema represents band offset between WO_3_ with a thin MoO*_z_*(S) film. CB and VB indicate the conduction band and valence band, respectively.

Figure 16 shows the results of a study into the energy band structures of MoO*_z_*(S)/WO_3_/FTO samples obtained by the anodic posttreatment of MoS_3.2_/WO_3_/FTO samples. The formation of a MoO*_z_*(S) film did not change the position of energy bands significantly. This position, however, is optimal for an efficient photoactivated OER process, which follows the traditional schema characteristic of Type II energy bands configuration (see Supplementary Materials Figure S12). Light absorption caused the generation of electron‒hole pairs in MoO_z_(S) and WO_3_ films. The preparation of the MoO*_z_*(S)/WO_3_ heterostructure made it possible to use the photogenerated holes in the MoO*_z_*(S) film quite efficiently, since the photogenerated in this film electrons sought to migrate into the WO_3_ film. The recombination of photogenerated electron‒hole pairs in MoO*_z_*(S) is slowed down due to migration of photogenerated electrons into the WO_3_ film. This is an energetically beneficial process since the lower edge of the conduction band in the latter film is below the lower edge of the conduction band in MoO*_z_*(S). Meanwhile, the photogenerated holes in these two semiconductors follow a reverse migration (see Supplementary Materials Figure S12). The WO_3_ film prevents a leakage of the photogenerated holes from MoO*_z_*(S) film into the WO_3_ film and provides an additional flux of the photogenerated in WO_3_ holes into the MoO*_z_*(S) film. Photogenerated holes move to MoO*_z_*(S) because of a higher energy of the upper edge of the valence band in MoO*_z_*(S). The holes accumulated in a MoO*_z_*(S) film can cause the enhanced efficiency of the OER process on the surface of MoO*_z_*(S)/WO_3_/FTO photoanode, provided the catalytic activity of the MoO*_z_*(S) film is higher than that of WO_3_.

It should be noted that the MoO*_z_*(S) film performs an important function, since it has a narrow energy band gap (~1.55 eV) and therefore effectively absorbs the Xe lamp radiation. For WO_3_ films with a moderate energy band gap (~2.7 eV), the theoretically achievable photocurrent density is ~4.5 mA/cm^2^ under AM1.5G illumination (100 mW/cm^2^) [15]. The use of an anodic semiconductor material with a narrow band gap allows this limit to be exceeded under several other conditions [68,69]. 

Note that the efficiency of MoO*_z_*(S)/WO_3_/FTO photoanodes depended on the composition of the initial MoS*_x_* precursor, which had undergone electrochemical posttreatment. The best properties were observed for the photoanodes obtained by post-treating MoS_3.2_/WO_3_/FTO samples. All the MoS*_x_*/WO_3_/FTO samples underwent anodic posttreatment in the same mode, which was determined by oxidation time and potential. Probably, obtaining efficient MoS*_x_*/WO_3_/FTO photoanodes requires each MoS*_x_* precursor to be post-treated differently.

## 5. Conclusions

Reactive PLD makes it possible to form consecutively nanostructured layers of the transition-metal oxide (WO_3_) and the transition-metal sulfide (MoS*_x_*) as well as to regulate their structural and chemical state by testing different substrate temperatures and reactive gas compositions and pressures. The optimization of the structural and chemical states of WO_3_ and MoS*_x_* thin films helps to obtain MoS*_x_*/WO_3_/FTO heterostructures on (FTO-coated) class substrates. Those structures have high efficiency in the photoelectrochemical process of HER in an acid solution. The best characteristics of photoactivated HER are associated with the samples that consist of porous nanocrystalline WO_3_ films (~250 nm thick) coated with porous amorphous MoS_4.5_ films (~100 nm thick) with a large effective area. On the MoS_4.5_/WO_3_/FTO photocathode, the HER photoelectrochemical process followed a Z-scheme mechanism, whereas the high efficiency of photoactivate HER was explained by an increased concentration of catalytically active states of sulfur. One cannot completely rule out the effect of sulfur concentration on the mechanisms of photoinduced charge transfer. In MoS_4.5_ films, the concentration of equilibrium holes, which could cause faster recombination with photogenerated electrons, was the lowest. The narrowest energy band gap of the MoS_4.5_ film facilitated the effective photogeneration of electron–hole pairs due to light absorption.

In MoS*_x_*/WO_3_/FTO heterostructures, MoS*_x_* films can be used as precursors to create effective MoO*_z_*(S)/WO_3_/FTO photoanodes for OER. To this end, it is important to select an optimal mode for the electrochemical posttreatment of MoS*_x_* films in order to obtain a MoO*_z_*(S) anodic material that is stable in an acid solution. A MoO*_z_*(S) film with relatively narrow energy band gap effectively absorbs light and ensures the migration of photogenerated holes to the surface of the MoO*_z_*(S)/WO_3_/FTO photoanode and causes OER to be catalyst-activated. 

## Supplementary Materials

The following are available online at https://www.mdpi.com/2079-4991/10/5/871/s1, Figure S1: RBS spectra for the WO_y_/FTO/glass samples. The WO_y_ films were obtained by RPLD at different pressures of dry air, Figure S2: SEM images of the WO_y_ films which were obtained in this work by reactive PLD at dry air pressures of (a) 20 and (b) 60 Pa. The temperature of the FTO substrate was 420 °C, Figure S3: VB-XPS spectrum of the WO_3_ film obtained by RPLD at a dry air pressure of 40 Pa, Figure S4: XPS Mo 3d and S 2p spectra for MoSx films, which were obtained by RPLD at different pressures of H_2_S gas, Figure S5: Tauc plots for the (a) MoS_2_ and (b) MoS_3.2_ films, Figure S6: VB-XPS spectra of relatively thick (a) MoS_2_, (b) MoS_3.2_, and (c) MoS_4.5_ films, Figure S7: (a) Tauc plot and (b) the leading edge of VB-XPS spectrum for thin MoOz(S) film, which was obtained on WO_3_ by electrochemical oxidation of precursor MoS_3.2_ film, Figure S8: Chronoamperometry stability measurement under chopped illumination of Xe lamp for the MoS_4.5_/WO_3_/FTO photocathode in 0.5 M H_2_SO_4_ solution at a potential of 0 V (RHE), Figure S9: Transmittance of the MoS_4.5_/WO_3_/FTO sample, Figure S10: Transient voltammogram of WO_3_/FTO and MoOz(S)/WO_3_/FTO photoanodes measured under a Xe lamp illumination at an applied potential of 1.6 V (RHE), Figure S11: The mechanism (Z-schema) of the photo-activated electrochemical process of HER (including the suggested mechanism of charge carrier transport) for the MoSx/WO_3_/FTO heterostructure. Band gaps and band edge positions for the heterostructure are shown, Figure S12: The mechanism of the photo-activated electrochemical process of OER (including the suggested mechanism of charge carrier transport) for the MoOz(S)/WO_3_/FTO heterostructure. Band gaps and band edge positions for the heterostructure are shown.
