# Supplementary material for: Performance and Mechanism of Photoelectrocatalytic Activity of MoSx/WO3 Heterostructures Obtained by Reactive Pulsed Laser Deposition for Water Splitting
Source: Nanomaterials (Basel). 2020 Apr 30;10(5):871. doi: 10.3390/nano10050871 (PMC7279322; doi:10.3390/nano10050871)
Supplement: Supplementary file 1 [file nanomaterials-10-00871-s001.pdf]

Supplementary Materials

Performance and Mechanism of Photoelectrocatalytic Activity of MoS_x/WO_3 Heterostructures Obtained by Reactive Pulsed Laser Deposition for Water Splitting

Vyacheslav Fominski [1,*], Roman Romanov [1], Dmitry Fominski [1], Alexey Soloviev [1], Oxana Rubinkovskaya [1], Maxim Demin [2], Ksenia Maksimova [2], Pavel Shvets [2] and Aleksandr Goikhman [2]

[1] National Research Nuclear University MEPhI (Moscow Engineering Physics Institute), Kashirskoe Sh. 31, Moscow 115409, Russia; limpo2003@mail.ru (R.R.); dmitryfominski@gmail.com (D.F.); ale7@inbox.lv (A.S.); oxygenofunt@gmail.com (O.R.)

[2] REC "Functional Nanomaterials", Immanuel Kant Baltic Federal University, Aleksandra Nevskogo St. 14, Kaliningrad 236041, Russia; mdemin@kantiana.ru (M.D.); xmaksimova@gmail.com (K.M.); pshvets@kantiana.ru (P.S.); aygoikhman@gmail.com (A.G.)

* Correspondence: vyfominskij@mephi.ru; Tel.: +7-903-242-2154

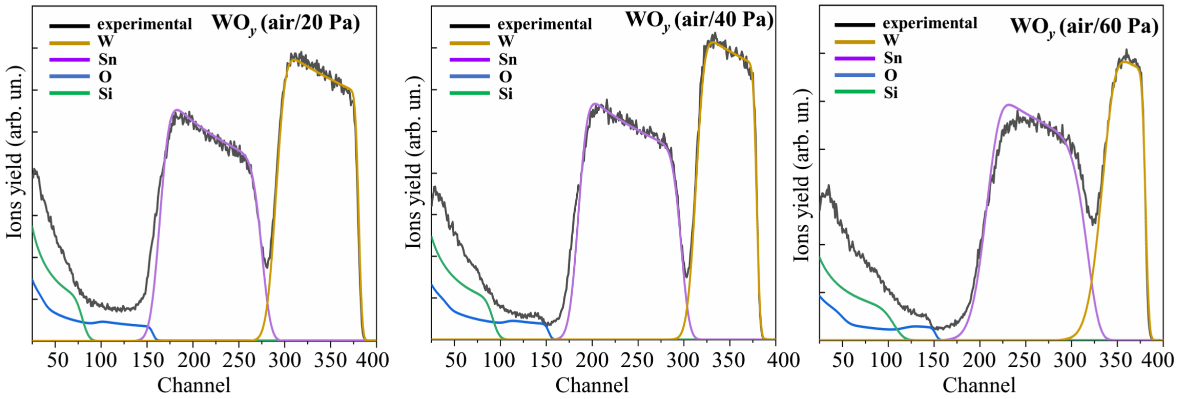

Figure S1. RBS spectra for the WO$_y$/FTO/glass samples. The WO$_y$ films were obtained by RPLD at different pressures of dry air. Colored lines indicated the spectra for different elements in these samples. Fluoride (in FTO layer) was not considered during decomposition of the spectra.

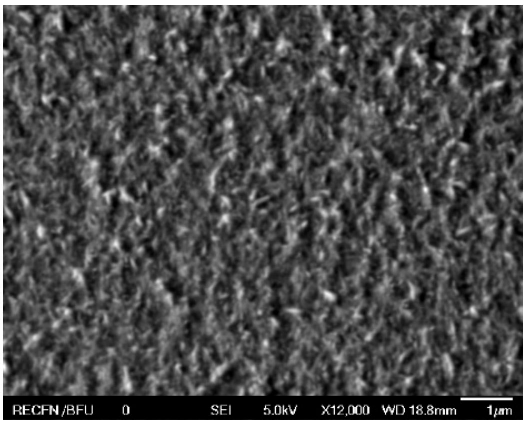

(**a**)

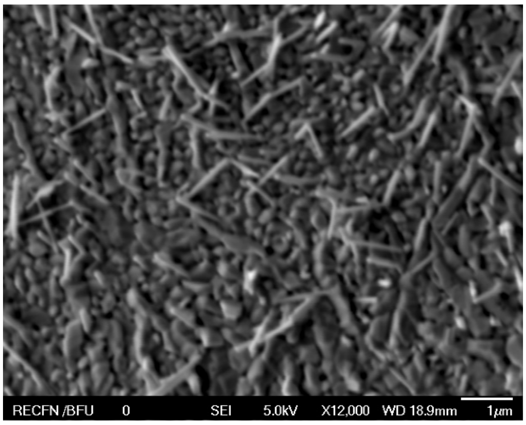

(**b**)

Figure S2. SEM images of the WO$_y$ films which were obtained in this work by reactive PLD at dry air pressures of (**a**) 20 and (**b**) 60 Pa. The temperature of the FTO substrate was 420 °C.

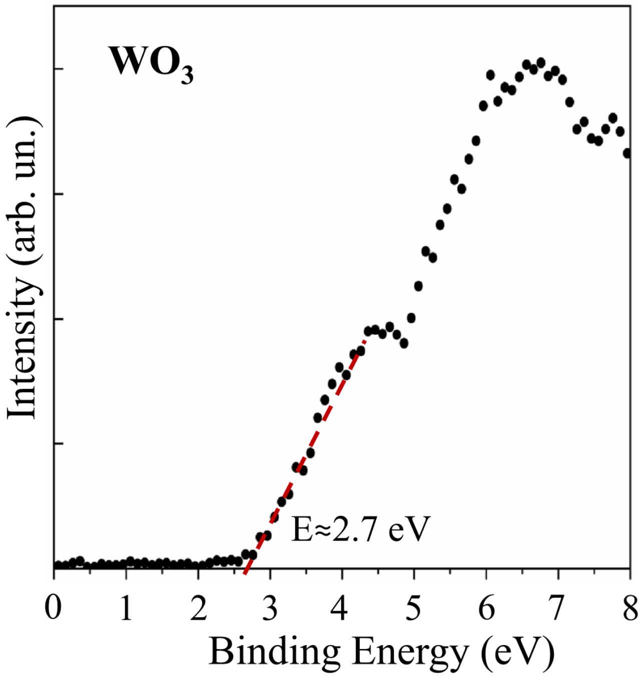

Figure S3. VB-XPS spectrum of the WO_3 film obtained by RPLD at a dry air pressure of 40 Pa.

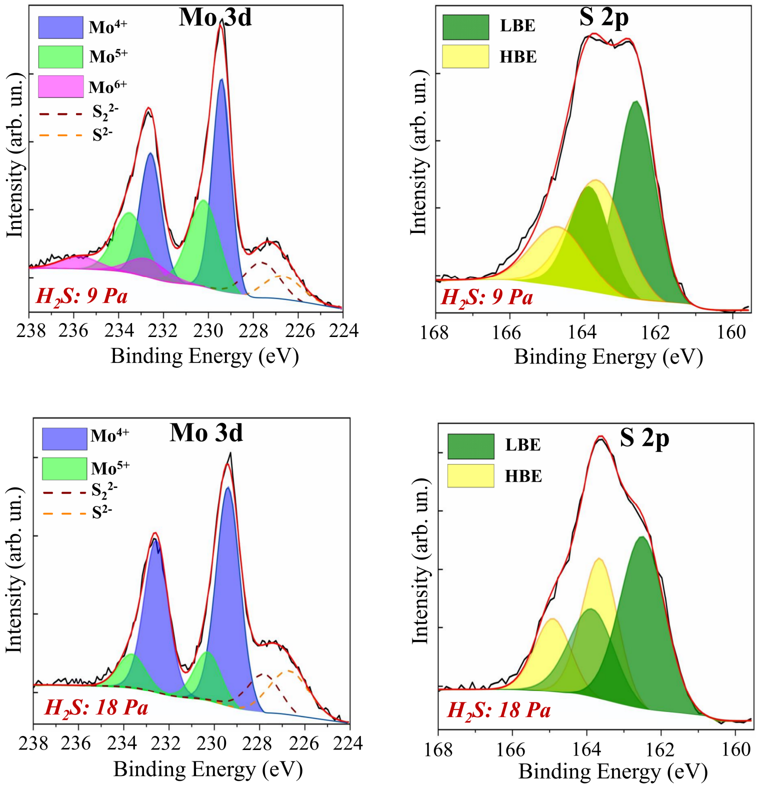

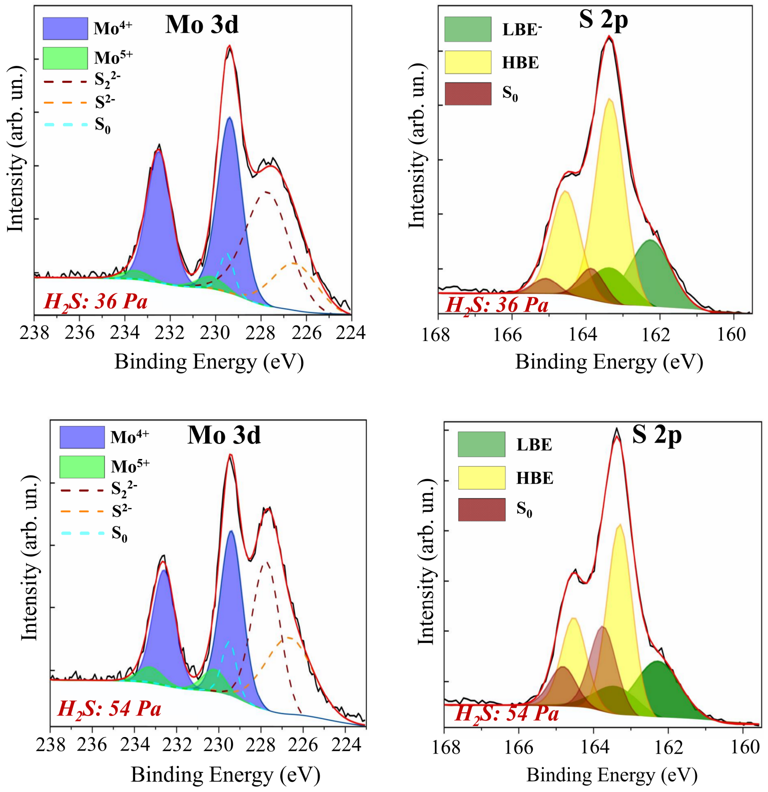

Figure S4. XPS Mo 3d and S 2p spectra for MoS_x films, which were obtained by RPLD at different pressures of H_2S gas.

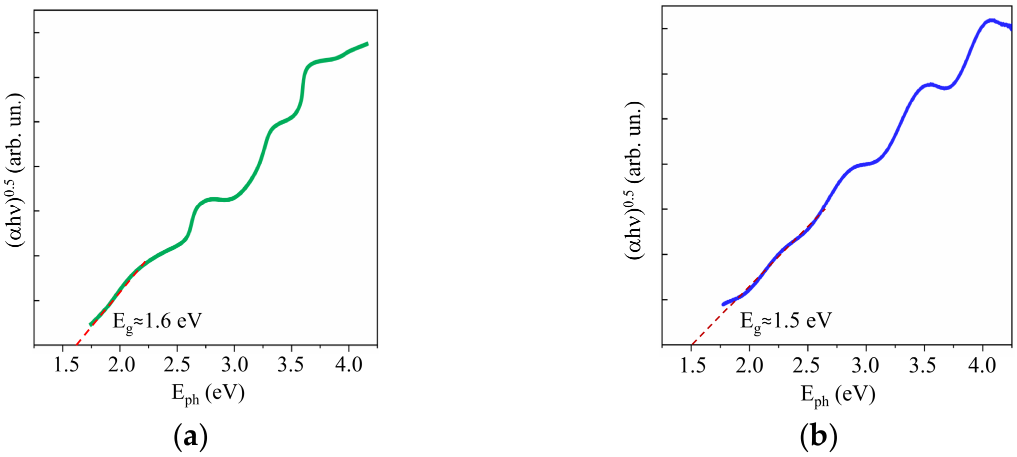

Figure S5. Tauc plots for the (**a**) MoS_2 and (**b**) $MoS_{3.2}$ films.

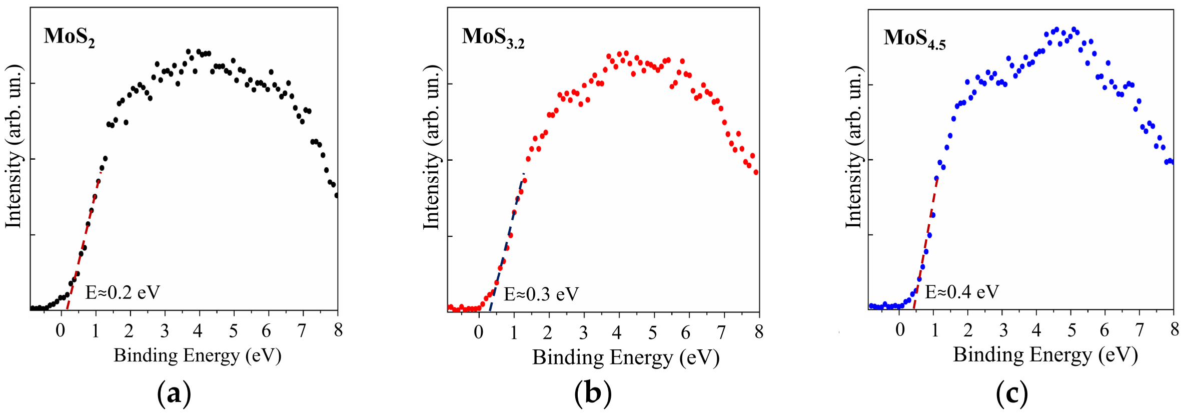

Figure S6. VB-XPS spectra of relatively thick (**a**) MoS_2, (**b**) $MoS_{3.2}$, and (**c**) $MoS_{4.5}$ films.

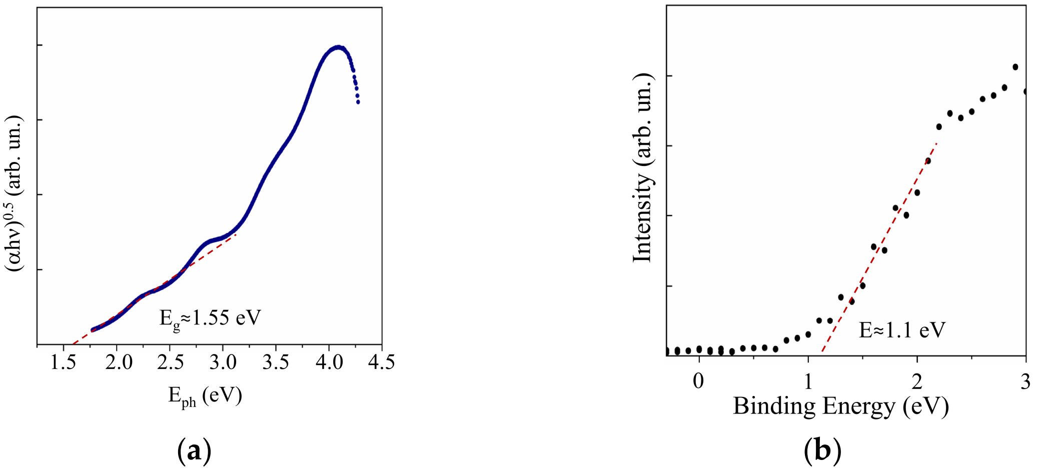

Figure S7. (**a**) Tauc plot and (**b**) the leading edge of VB-XPS spectrum for thin MoO_z(S) film, which was obtained on WO_3 by electrochemical oxidation of precursor $MoS_{3.2}$ film.

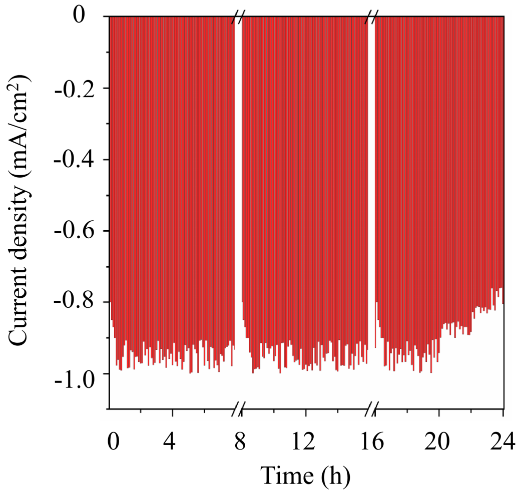

Figure S8. Chronoamperometry stability measurement under chopped illumination of Xe lamp for the $MoS_{4.5}/WO_3/FTO$ photocathode in 0.5 M H_2SO_4 solution at a potential of 0 V (RHE). The measurement was carried out for 3 days (during 8 h per day).

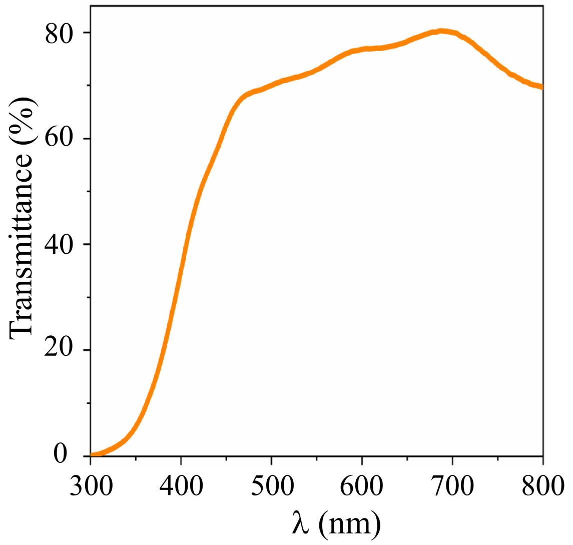

Figure S9. Transmittance of the $MoS_{4.5}/WO_3/FTO$ sample.

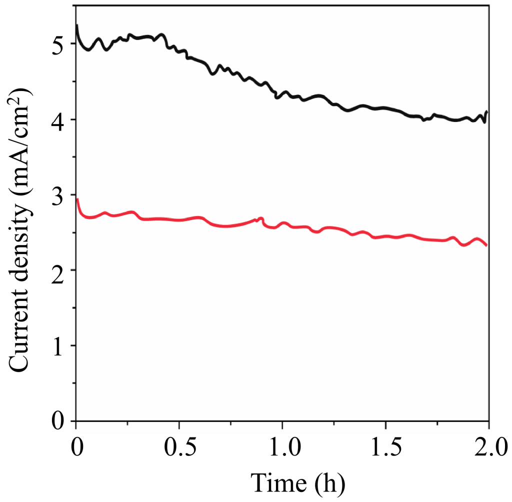

Figure S10. Transient voltammogram of WO_3/FTO and MoO_z(S)/WO_3/FTO photoanodes measured under a Xe lamp illumination at an applied potential of 1.6 V (RHE).

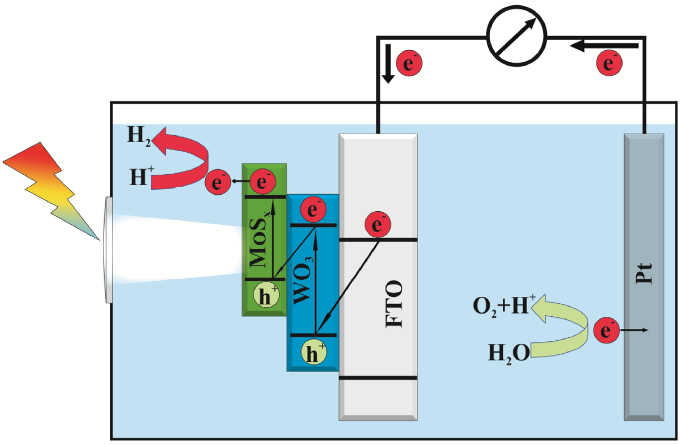

Figure S11. The mechanism (Z-schema) of the photo-activated electrochemical process of HER (including the suggested mechanism of charge carrier transport) for the MoS_x/WO_3/FTO heterostructure. Band gaps and band edge positions for the heterostructure are shown.

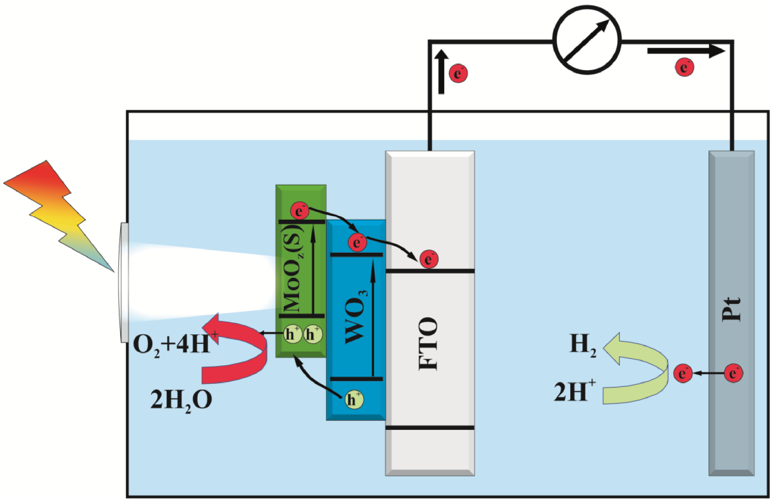

Figure S12. The mechanism of the photo-activated electrochemical process of OER (including the suggested mechanism of charge carrier transport) for the $MoO_2(S)/WO_3/FTO$ heterostructure. Band gaps and band edge positions for the heterostructure are shown.
